# Resistant Sources and Genetic Control of Resistance to ToLCNDV in Cucumber

**DOI:** 10.3390/microorganisms9050913

**Published:** 2021-04-24

**Authors:** Cristina Sáez, Laura G. M. Ambrosio, Silvia M. Miguel, José Vicente Valcárcel, María José Díez, Belén Picó, Carmelo López

**Affiliations:** Instituto de Conservación y Mejora de la Agrodiversidad Valenciana, Universitat Politècnica de València, Avenida de los Naranjos s/n, 46022 Valencia, Spain; lauragma97@gmail.com (L.G.M.A.); silviammiguelmontero@gmail.com (S.M.M.); jvalcarc@btc.upv.es (J.V.V.); mdiezni@btc.upv.es (M.J.D.)

**Keywords:** *Begomovirus*, cucumber, mechanical inoculation, real-time PCR, viral load, QTLs, resistance

## Abstract

Tomato leaf curl New Delhi virus (ToLCNDV) is a severe threat for cucurbit production worldwide. Resistance has been reported in several crops, but at present, there are no described accessions with resistance to ToLCNDV in cucumber (*Cucumis sativus*). *C. sativus* var. *sativus* accessions were mechanically inoculated with ToLCNDV and screened for resistance, by scoring symptom severity, tissue printing, and PCR (conventional and quantitative). Severe symptoms and high load of viral DNA were found in plants of a nuclear collection of Spanish landraces and in accessions of *C. sativus* from different geographical origins. Three Indian accessions (CGN23089, CGN23423, and CGN23633) were highly resistant to the mechanical inoculation, as well as all plants of their progenies obtained by selfing. To study the inheritance of the resistance to ToLCNDV, plants of the CGN23089 accession were crossed with the susceptible accession BGV011742, and F_1_ hybrids were used to construct segregating populations (F_2_ and backcrosses), which were mechanically inoculated and evaluated for symptom development and viral load by qPCR. The analysis of the genetic control fit with a recessive monogenic inheritance model, and after genotyping with SNPs distributed along the *C. sativus* genome, a QTL associated with ToLCNDV resistance was identified in chromosome 2 of cucumber.

## 1. Introduction

Cucurbits are cultivated in tropical, subtropical, and temperate regions of the New and Old world and supply essential vitamins and minerals to current diets in countries around the world, being a major source of food for humans. Crops belonging to the three most economically important genera, *Cucumis* (melon and cucumber), *Citrullus* (watermelon), and *Cucurbita* (zucchini, pumpkin, squash and gourd), rank in the first positions in global vegetable and fruit production. Spain is one of the main world producers of cucurbits [1], and the first exporting country in Europe. However, the production of these crops has been severely affected by diseases, in particular those caused by viruses [2,3] that have a high economic impact. Among them, *Tomato leaf curl New Delhi virus* (ToLCNDV), a member of the genus *Begomovirus*, family *Geminiviridae*, has spread rapidly in southern Spain since the first detection in 2012 and represents a major risk in the production of zucchini, melon, and cucumber.

ToLCNDV was first detected in tomato (*Solanum lycopersicum* L.) in India in 1995 [4] and, later, it was found in other south and southeast Asian countries in several hosts, particularly species of the *Solanaceae* and *Cucurbitaceae* families [5,6]. ToLCNDV was limited to Asian countries until 2012, when it was reported affecting cucurbits (mainly zucchini (*Cucurbita pepo* L.), melon (*Cucumis melo* L.), and cucumber (*Cucumis sativus* L.)) in different Mediterranean countries, first in Spain and later in Tunisia, Italy, Morocco, Greece, and Algeria [7,8,9,10,11,12]. More recently, the virus has been identified in cucurbit plants in Portugal and Estonia [13], and in species of the *Solanaceae* family in Italy [14], so ToLCNDV is rapidly spreading through Europe. 

ToLCNDV consists of two circular single-stranded DNA molecules of approximately 2.7 kb each (designated as DNA-A and DNA-B) [15]. The symptoms caused by ToLCNDV depend on the species and the time of infection, but it usually induces curling, leaf mottling and mosaic of young leaves, short internodes, and fruit skin roughness [7], often resulting in a significant yield reduction. ToLCNDV is naturally transmitted by the whitefly *Bemisia tabaci* (Gennadius) byotipes MED and MEAM1 in a persistent manner [15,16,17] although some isolates are also mechanically sap-transmitted to different hosts [16,18,19]. Recently, seed-transmissible strains of ToLCNDV have been described infecting chayote (*Sechium edule* (Jacq) Sw) in India [20], and zucchini squash in Italy [21]. Against this background, the European and Mediterranean Plant Protection Organization (EPPO) included this virus in the EPPO Alert List [22].

ToLCNDV is currently managed using cultural practices and chemical treatment against its vector. However, these control methods have limited effectiveness and can be expensive. Therefore, the development of resistant varieties through conventional breeding provides an effective and sustainable solution for reducing the impact of the disease caused by this virus. In cucurbits, monogenic resistance to ToLCNDV has been described in sponge gourd (*Luffa cylindrica* M. Roem.) [17,23]. In melon, resistance has been identified in five Indian melon genotypes belonging to subsp. *agrestis* (Naudin) Pangalo (three accessions of the *momordica* horticultural group and two wild *agrestis*) [19]. A major QTL in chromosome 11 was found controlling the resistance to ToLCNDV in one of the wild *agrestis* accessions, with epistatic interactions of two additional regions in chromosomes 2 and 12 [24]. Finally, resistance has also been identified in pumpkin (*Cucurbita moschata* L.) accessions from diverse origins [25]. A major recessive gene located in chromosome 8, in a region syntenic to the candidate region in chromosome 11 of melon, was found controlling the resistance to ToLCNDV in this species [26]. 

The first step for breeding resistant cucumber cultivars is the search for resistant sources. Cucumber germplasm has been screened for resistance to different viral diseases, but to our knowledge, no resistance has been described for ToLCNDV in cucumber [3]. In this report, we evaluated the response to ToLCNDV of a cucumber germplasm collection by mechanical inoculation. The identification of three Indian *C. sativus* accessions highly resistant to the virus, which remained symptomless and showed a reduced viral accumulation, provides the first sources for breeding ToLCNDV-resistant cucumber cultivars. Moreover, we have identified one QTL controlling the resistance to ToLCNDV in *C. sativus* using segregating populations derived from one of these resistant sources and a susceptible accession.

## 2. Materials and Methods

### 2.1. Plant Material

A nuclear collection of 40 Spanish landraces of *C. sativus* var. *sativus* (Table 1), held at the genebank of the Institute for the Conservation and Breeding of Agricultural Biodiversity at the Polytechnic University of Valencia (COMAV-UPV), was first screened in a climatic chamber against ToLCNDV by mechanical inoculation. These accessions represent the variability of the full COMAV collection, consisting of 217 accessions collected from diverse Spanish origins and multiplied by COMAV [27,28]. This collection includes accessions belonging to the typical “short” (20) “long” (16), and “French” (4) cucumber types (Table 1), which are highly appreciated on national and international markets because of their quality. Additionally, 23 *C. sativus* var. *sativus* accessions from different geographical origins (Table 2) of the “short” (12), “medium” (5), and “long” (5) cucumber types, and one unknown type, were also tested. Seeds of these accessions were firstly provided by the Centre for Genetic Resources (CGN germplasm collection, the Netherlands), and then multiplied at COMAV.

### 2.2. Virus Source, Mechanical Inoculation, and Symptom Evaluation

As an inoculum source, zucchini plants of the MU-CU-16 accession were agroinoculated by injection into petioles with an infectious clone of ToLCNDV [25]. ToLCNDV transmission to cucumber plants was performed by mechanical inoculation at the stage of one true leaf, as described by López et al. [19]. Briefly, inoculum was prepared by grinding 1 g of symptomatic leaf tissue from agroinfiltrated plants in the presence of inoculation buffer in a 1:4 (*w*:*v*) proportion. The expanded true leaf and one cotyledon of each plant were dusted with carborundum (600 mesh) and then inoculated by rubbing with a cotton-bud stick, gently soaked in the crude homogenized inoculum.

**Figure 1:**
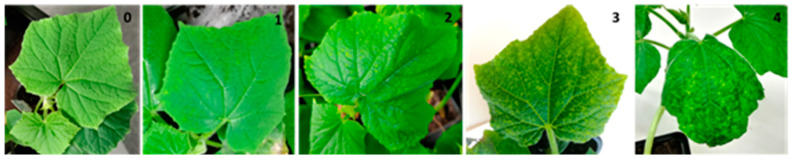
Symptom scoring in cucumber plants showing ToLCNDV symptoms corresponding to the scale: 0 absence of symptoms, 1: mild symptoms, 2: moderate symptoms, 3: severe symptoms, and 4: very severe symptoms or dead plant.

For the mechanical inoculation, seeds were disinfected in a 10% solution of sodium hypochlorite for 3 min and washed for 5 min in distilled water. Germination was performed in Petri plates with moistened cotton at 37 °C for 48 h. Seedlings were transplanted to pots in a growth chamber under a photoperiod of 16 h day at 25 °C and 8 h night at 18 °C and 70% relative humidity. Seedlings at the one true leaf stage were mechanically inoculated, leaving two uninoculated plants per genotype as controls. Inoculated plants were individually evaluated at 15 and 30 days post inoculation (dpi) for the presence and severity of virus symptoms. Symptoms on upper leaves were recorded by visual evaluation using the following scale: 0, no symptoms; 1, mild symptoms; 2, moderate symptoms; 3, severe symptoms; 4, very severe symptoms or dead plant (Figure 1). Additionally, every plant was assayed for the presence of virus using the tissue printing technique and conventional PCR with the protocols described below. Additionally, the viral load of ToLCNDV was determined by qPCR in a selected number of accessions with the best resistance response (CGN22297, CGN22986, CGN23089, CGN23423, and CGN23633). The number of plants tested of each accession varied between 3 and 6 due to seed availability and germination. The most resistant accessions were selected for further analysis with additional plants.

### 2.3. ToLCNDV Detection by Tissue Printing 

For detection of ToLCNDV in tissue prints, plant petioles of the upper leaves at 15 dpi were cut with a razor blade and cross-sections were blotted onto positively charged nylon membranes (Hybond-N, Amersham) immediately after cutting. Membranes were air dried, fixed by UV irradiation (700 × 100 mJ/cm^2^), and hybridized with a digoxigenin-labeled RNA probe. The riboprobe was generated by transcription with T7 RNA polymerase from a recombinant pTZ57R plasmid (Fermentas) with an insert corresponding to the complete CP gene of ToLCNDV in a negative orientation, following the manufacturer’s instructions (Roche Diagnostics). Prehybridization, hybridization, and washing of the membranes were performed as previously reported [29], except that the hybridization was conducted at 60 °C. Chemiluminiscent detection using CSPD reagent as substrate was performed as recommended by the manufacturer (Roche Diagnostics). Films were exposed to the membranes at room temperature for 30–60 min. 

### 2.4. ToLCNDV Detection by PCR and qPCR

To confirm the presence of the virus by PCR, total DNA from apical leaves of inoculated and control plants was extracted at 30 dpi using the CTAB method [30]. DNA was quantified using a NanoDrop 1000 spectrophotometer (Thermo Scientific) and diluted with sterile deionized water to a final concentration of 50 ng μL^−1^. One-microliter aliquots of total DNA (50 ng) were used as templates in PCR reactions with the ToLCNDV-specific primer pairs To-A1F and To-A1R from DNA-A [25]. The resulting PCR products of 504 bp in length were analyzed by electrophoresis in 1.0% agarose gels in TAE buffer.

The relative ToLCNDV accumulation in individual plants of the most promising accessions was determined at 30 dpi by qPCR, and susceptible plants were used as controls. DNA was diluted to a final concentration of 5 ng μL^−1^ and all plants were analyzed in two technical replicates using a *LightCycler*^®^ 480 System (Roche). In each qPCR reaction, 15 ng of genomic DNA were used as templates, in a final volume of 10 μL. We used 2.5 μL of MasterMix qPCR No-ROX PyroTaq EvaGreen 5x (Cmb bioline) and 0.35 μL (10 µM) of each primer and 3.8 μL of H_2_O. Primers ToLCNDVF1 (5′-AATGCCGACTACACCAAGCAT-3′, positions 1145–1169) and ToLCNDVR1 (5′-GGATCGAGCAGAGAGTGGCG-3′, positions 1399–1418), derived from the Spanish isolate Murcia 11.1 (segment DNA-A, accession number KF749225), were used for the amplification of a 273 bp fragment of viral DNA-A. The *β-actin* of *C. sativus* gene was amplified in all samples as reference control using an adapted design of primers used in previous works on watermelon [31], ClACT-F (5′-CCATGTATGTTGCCATTCAG-3′) and ClACT-R (5′-GGATAGCATGGGGAAGAGCA-3′). Cycling conditions consisted of incubation at 95 °C for 15 min, 45 cycles of 95 °C for 5 s, 60 °C for 30 s, and 72 °C for 30 s. Relative ToLCNDV levels were calculated using the 2^−ΔΔCt^ expression of the Livak method [32], where ΔΔCt is the difference between the ΔCt of each sample and the ΔCt of the calibrator sample.

### 2.5. Validation of Response to the Viral Infection and Generation of F_1_, F_2_, and BC Populations 

Plants of each of the three resistant accessions were transplanted, grown, and selfed in a whitefly-proof greenhouse and the plant with the best resistant behavior, CGN23089-2, was crossed with plants of the accession BGV011742, highly susceptible to ToLCNDV. Seventeen seeds of the F_1_ hybrid and from 15 to 20 seeds of the selfing offspring of each parent were disinfected and seedlings were transplanted to pots and grown in a climatic chamber under controlled conditions. All plants were mechanically inoculated with ToLCNDV and phenotyped according to symptomatology and viral accumulation determined by qPCR, at 15 and 30 dpi, following the procedure described above. Three plants of the genotype BGV011742 were also included as susceptible controls in the validation assay. Means of 2^(−ΔΔCt)^ values of each genotype were analyzed by analysis of variance (ANOVA) and least significance difference (LSD) multiple range tests using STATGRAPHIC 18 ^TM^ (Statgraphics Technologies, Inc., The Plains, VA, USA), to evaluate statistically significant differences between them, with a level of confidence of 95%.

In the following growing season, three plants of the F_1_ progeny were cultivated in a greenhouse to generate F_2_ segregating populations by selfing, and BC_CGN23089_ and BC_BGV011742_ by backcrossing to plants of CGN23089 and BGV011742, respectively. All plants of these populations were screened against ToLCNDV with the same inoculation protocol and disease assessment procedure described above. The chi-squared (*χ*^2^) test (*p* < 0.05) was used to determine the goodness of fit between the expected and observed ratios of resistant:susceptible segregation in the three populations. 

### 2.6. Genotyping of Segregating Populations

To genotype the segregating populations, single nucleotide polymorphism (SNP) markers were selected from two sources: a genotyping-by-sequencing (GBS) assay, including the BGV011742 accession, performed in previous studies by our group, and the available data of a GBS assay used to characterize the United States National Plant Germplasm System (NPGS) collection of cucumber [33]. Among the analyzed accessions, the Indian genotype PI 197087 is the same as the accession CGN23089 kept in the CGN germplasm collection, as described in the passport data from both genebanks (https://cgngenis.wur.nl (accessed on 28 February 2021); https://www.ars-grin.gov/ (accessed on 14 February 2021)). Both sequences were aligned against the cucumber genome Gy14 v.2 available in the Cucurbit Genomics Database (http://cucurbitgenomics.org; (accessed on 15 March 2021)) using the Bowtie2 tool [34], and SNP variants were found by Freebayes version 1.0.2 [35]. A panel of 47 SNPs was designed to cover the seven chromosomes of the cucumber genome and used to genotype parents, F_2_, and BC segregating populations by an Agena Bioscience iPLEX^®^ Gold MassARRAY (Agena Biosciences, CA, USA) system at the Epigenetic and Genotyping Unit of the University of Valencia (Unitat Central d’Investigació en Medicina (UCIM), Faculty of Medicine, Malaga, Spain). F_2_ genotyping results were used to construct a genetic map using the Kosambi map function in MAPMAKER 3.0 [36], and a QTL analysis was performed applying the composite interval mapping approach (CIM) in Qgene 4.0 [37]. Symptom score, ToLCNDV relative accumulation at 30 dpi, and a qualitative trait of resistance, assigning to each plant a category of 0 if the phenotype was susceptible and 1 if it was resistant, were used to identify markers linked to the resistance to ToLCNDV. LOD threshold was estimated performing 1000 permutation tests per trait, with *p* < 0.05. The proportion of phenotypic variance explained (*R*^2^), the additive and dominance effects, and the interval position of the QTL, according to a LOD drop of up to the significant LOD threshold level, were estimated for the peak LOD of each significant QTL. Since these traits were not normally distributed, we used a Kruskal–Wallis non-parametric test to support CIM QTL detection, using MapQTL version 4.1 software [38], considering associations significant at *p* ≤ 0.05.

Means of symptom scores and 2^(−ΔΔCt)^ at 30 dpi of BC_CGN23089_ plants were calculated for the closest SNPs to the QTL peak LOD, according to each genotypic class (b and h). To determine statistically significant differences between means (*p* ≤ 0.05), ANOVA and LSD multiple range tests were performed using STATGRAPHIC 18^TM^ (Statgraphics Technologies, Inc., The Plains, VA, USA) statistical software.

## 3. Results

### 3.1. Response of the Spanish Landraces of C. sativus to the Mechanical Transmission of ToLCNDV 

A core collection of 40 accessions from different Spanish provinces held at the COMAV Genebank was assayed. Most of the 40 tested cucumber accessions were highly susceptible to the mechanical transmission of ToLCNDV, showing moderate symptoms of mottling to severe symptoms characterized by mosaic and yellowing of young leaves (Figure 1). Symptoms started to appear at different days after mechanical inoculation. On average, symptom scores in plants of the “short”, “long”, and “French” types of *C. sativus* increased from 15 to 30 dpi, with average scores from 1.78 ± 0.19 to 2.1 ± 0.2, 1.52 ± 0.19 to 1.9 ± 0.19, and 2.43 ± 0.31 to 2.8 ± 0.31 in each group, respectively, in a range of 0 to 4 (Table 1). As had already been observed in the cucumber-growing areas of the southeast of Spain, plants of the “French” type were the most susceptible [39].

To further characterize the response to ToLCNDV, the viral load of all plants at 15 dpi was evaluated by molecular hybridization by tissue printing. To carry out a more precise confirmation, viral accumulation was determined by semi-quantitative PCR at 30 dpi. Viral load ranged from intermediate to high (Table 1) in most of the genotypes at 15 dpi. In addition, similar high ToLCNDV titers were detected by PCR at 30 dpi in all genotypes (data not shown). Only accessions BGV000479, BGV002495, BGV002494, and BGV004851 developed mild symptoms at the end of the trial, although with moderate or high viral accumulation. On average, most of the plants of the BGV011586 accession displayed low symptomatology and accumulated low viral titers, but some plants developed high symptoms from the beginning of the assay, suggesting variability in the response to ToLCNDV within this genotype.

### 3.2. Response of the C. sativus Accessions from Different Origins to the Mechanical Transmission of ToLCNDV

The cucumber accessions from different countries showed variable responses to ToLCNDV infection. Susceptible accessions behaved similarly to the Spanish landraces, displaying moderate to severe yellowing and mottling that in most cases increased from 15 to 30 dpi. Accessions belonging to the “long” type, all originating from China, had on average higher symptom scores (Table 2) at 15 dpi, with a mean of 1.86 ± 0.2. Similarly, the only accession of unknown type (CGN19655, originating from the U.S.A.) was highly susceptible, with a symptom score of 1.4 at 15 dpi (Table 2). Viral titers detected with PCR were high or very high in all these accessions (data not shown).

Interestingly, lower severity of the ToLCNDV infection was observed in some Indian genotypes of the “medium” and “short” types. Accession CGN22297 was symptomless or had very mild symptoms at 15 dpi (mean symptom score of 0.4, in a range between 0 and 1), although some plants developed moderate symptomatology at the end of the assay (plants with symptom scores ranging from 0 to 2) (Table 2). All plants of accessions CGN22297, CGN22986, CGN23089, CGN23423, and CGN23633 remained symptomless, or had symptom scores lower than one, throughout the screening assay. On these five accessions, ToLCNDV titers were low or not detected by probe hybridization at 15 dpi (Table 2), although in all of them the virus was detected at 30 dpi after PCR analysis.

Among the remaining “medium” and “short” type assayed accessions from different countries, some initially had a promising behavior (mean symptom score 1.14 and 1.36, respectively, ranging from 0.3 to 3.5), but typical severe ToLCNDV symptomatology and high or very high viral titers were identified in all of them at different stages of the disease (Table 2).

### 3.3. ToLCNDV Quantification in Resistant Genotypes

**Figure 2:**
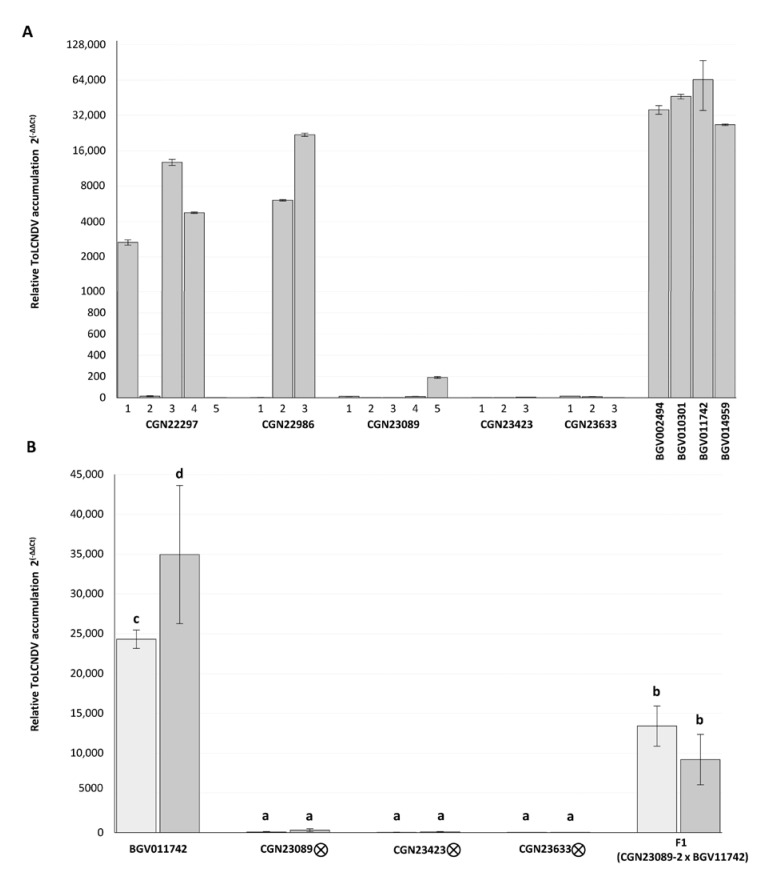
(**A**) Relative ToLC NDV accumulation (2^(−ΔΔCt)^) at 30 days after mechanical inoculation (dpi) with ToLCNDV in the five asymptomatic Indian accessions (CGN22297, CGN22986, CGN23089, CGN23423, and CGN23633) and in four susceptible controls (BGV002494, BGV010301, BGV011742, and BGV014959). (**B**) Relative ToLCNDV accumulation (2^(−ΔΔCt)^) at 15 and 30 dpi (light and dark bars, respectively) of plants obtained by selfing the CGN23089-3, CGN23423-2, and CGN23623-2 genotypes and of the F1 (CGN23089-2 x BGV011742) hybrids. On the *x* axis, accessions and number of plants of each accession are indicated. Bars sharing the same letter are not significantly different, according to ANOVA and LSD tests (*p ≤* 0.05).

Individual plants of the five Indian accessions with better response after infection with ToLCNDV were tested by qPCR to further determine the viral accumulation at 30 dpi. One or two plants of the susceptible accessions BGV002494, BGV010301, BGV011742, and BGV014959 were used as controls, with all of them showing the highest level of relative viral titers, but the resistant genotypes presented variability between their relative ToLCNDV accumulations (Figure 2A). Plants of the CGN23089, CGN23423, and CGN23633 accessions had uniformly low viral loads, with 2^(−ΔΔCt)^ values 1.9 × 10^3^ times lower than the levels accumulated by the susceptible plants, on average. Instead, in both CGN22297 and CGN22986 accessions, some plants were identified with low 2^(−ΔΔCt)^ values and some with high viral load, similar to that detected in one of the susceptible genotypes (Figure 2A). After this further characterization, the accessions CGN23089, CGN23423, and CGN23623, which were those with the lowest symptoms scores at 15 and 30 dpi and with the lowest viral titers estimated with different methods, were selected for further characterization.

### 3.4. Response of Self-Pollinated and F_1_ Progenies to the Mechanical Transmission of ToLCNDV

**Figure 3:**
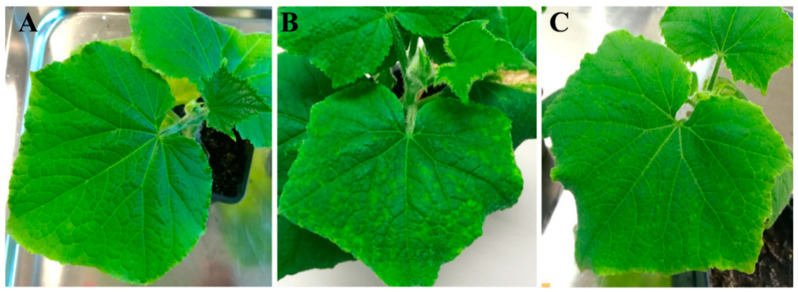
(**A**) Asymptomatic plant of the resistant accession CGN23089 30 days after mechanical inoculation (dpi) with ToLCNDV. (**B**) Symptomatic plant of the susceptible accession BGV011742. (**C**) Symptoms in an F_1_ plant of the cross CGN23089 x BGV011742 at 30 dpi.

The selfing offspring of the plants CGN23089-3, CGN23423-2, and CGN23623-2, and the F_1_ hybrid derived from the cross CGN23089-2 x BGV011742 (one of the most susceptible Spanish landraces selected as a susceptible parent for this cross), were mechanically inoculated with ToLCNDV in a second assay to confirm the resistance. As expected, plants of the susceptible BGV011742 parent showed severe symptoms at 30 dpi (Figure 3B), while all plants of the self-pollinated offspring had a similar behavior to that observed in the resistant plants of the first assay, displaying mild to no symptoms at 15 and 30 dpi (Figure 3A).

The F_1_ (CGN23089-2 x BGV011742) plants developed moderate symptomatology (two on the symptom scale) at 15 dpi and the same behavior was observed up to the end of the assay (Figure 3C). On average, viral titer in the F_1_ hybrid surpassed those of the CGN23089, CGN23423, and CGN23633 accessions by more than one hundred times, but it was similar to the high viral accumulation detected in some plants of the CGN22297 and CGN22986 accessions. Nevertheless, the average viral load in the F_1_ hybrid was almost three times lower than in the susceptible accessions (Figure 2B).

### 3.5. Response of Segregating Populations to the Mechanical Transmission of ToLCNDV

**Figure 4:**
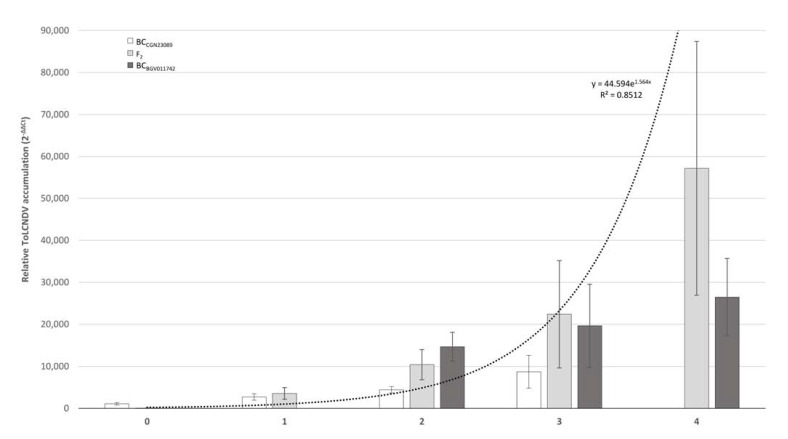
Mean of relative viral accumulation (2^−ΔΔCt^) at 30 days after mechanical inoculation in plants of F_2_ (CGN23089-2 x BGV011742) (light gray bars), BC_CGN23089_ (white bars), and BC_BGV011742_ (dark gray bars) in each symptom score category. Dotted line shows the tendency of the variable adjusted to an exponential model.

After ToLCNDV mechanical inoculation, both F_2_ and BC_CGN23089_ segregated for symptom development and viral load, while all assayed plants of BC_BGV011742_ developed severe symptoms and high viral accumulation. The number of resistant and susceptible plants found in each segregating population is shown in Table 3, according to symptomatology and viral load at 30 dpi. At the end of the assay, 31 plants of F_2_ remained symptomless or had slight symptoms (scores 0 to 1), and 65 showed moderate to severe symptomatology (scores 2 to 4). This segregation fit an expected ratio of 1:3 (resistant:susceptible), compatible with a single recessive gene controlling the resistance (*p* = 0.099) (Table 3). On average, viral accumulation correlated to symptom severity following an exponential model (y = 44.594e^1.564x^, *R*^2^ = 0.8512), with 2^(−ΔΔCt)^ viral load values of up to 10^4^ times higher in susceptible plants than in resistant plants (Figure 4). In BC_CGN23089_, 21 plants were resistant (scores 0 to 1) and 33 were susceptible (scores 2 to 3), fitting a 1:1 expected segregation for recessive monogenic control (*p* = 0.1025) (Table 3). Within each symptom score category, plants of both BC populations accumulated similar viral titers (Figure 4).

### 3.6. Genotyping and Linkage Analysis in Segregating Populations

After genotyping the F_2_ and BC populations with the 47 SNP markers evenly distributed throughout the *C. sativus* genome, only 18 SNPs were polymorphic between the CGN23089 and BGV011742 accessions. Genotypic results of F_2_ were used to construct a linkage map of the seven chromosomes, spanning a total of 554 cM of genetic distance with an average of 34.67 cM between markers (Table S1).

**Figure 5:**
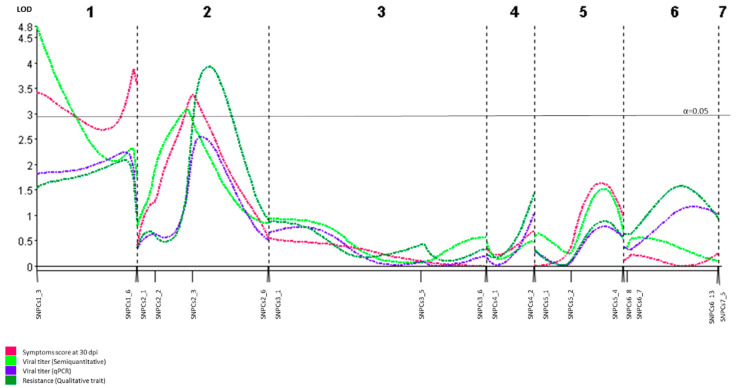
QTL analysis of F_2_ (CGN23089-2 x BGV011742) using symptom score at 30 days after mechanical inoculation (dpi), viral titers of ToLCNDV at 30 dpi (semiquantitative and quantitative detection) and qualitative resistance as traits.

To identify genomic regions linked to the resistance to ToLCNDV in cucumber, a QTL analysis was performed. Symptoms at 30 dpi, viral accumulation at 30 dpi determined by probe hybridization, and the qualitative trait of resistance showed significant association with three overlapping QTLs in chromosome 2, explaining between 15.1 and 17.3% of the observed phenotypic variance (Table 4). A fourth QTL was linked to viral accumulation determined by qPCR (ΔΔCt), but the LOD peak obtained (2.54) was slightly under the LOD threshold (2.75) (Figure 5). The closest marker to all significant QTLs (ToLCNDVCs_Sy30-2, ToLCNDVCs_VT30-2, and ToLCNDVCs_Re-2) was SNPCS2_3 (physical position 12,760,375 pb), with LOD peaks between 3.07 and 3.93. All these QTLs were statistically validated by a Kruskal–Wallis test, with *p* ≤ 0.005. Two additional QTLs were identified in chromosome 1 (Figure 5), but their effects were not significant on all the traits. Thus, they were excluded from the analysis. According to the regions with a significant LOD value, the interval of the QTL was delimited between 11,657,498 pb and 21,993,369 pb genomic positions.

**Figure 6:**
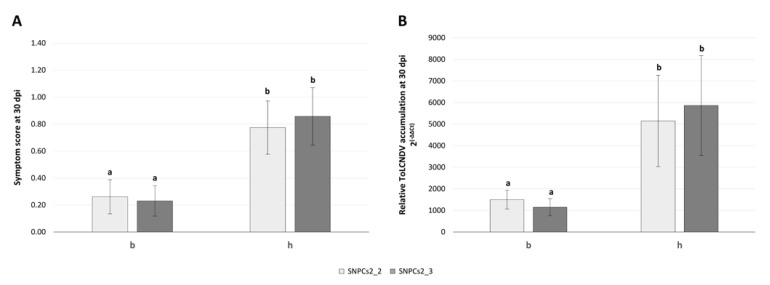
Mean of symptom score (**A**) and relative viral accumulation (**B**) at 30 days after mechanical inoculation in BC_BGV011742_ according to each genotypic class of SNPCs2_2 and SNPCs2_3 markers (chromosome 2). On the *x* axis, homozygous genotype of CGN23089 allele is represented as “b”, heterozygous genotype is represented as “h”. Bars with same letter are not significantly different at *p* ≤ 0.05.

To validate the effect of the chromosome 2 region in the BC_CGN23089_ population, the mean of symptom scores and relative viral accumulation at 30 dpi were calculated for each genotypic class of the two closest SNPs to the identified QTL interval (SNPCs2_2 and SNPCs2_3). The lowest level of symptoms and viral load was observed in plants with a homozygous genotype (b) for both markers, while heterozygous (h) plants for any of these markers showed more severe symptomatology and accumulated more ToLCNDV particles (Figure 6).

## 4. Discussion

Forty accessions of cucumber collected from different provinces of Spain were screened, in order to find sources of resistance against ToLCNDV, but none of the accessions showed immunity or high resistance to the virus. Most accessions were highly susceptible after ToLCNDV mechanical inoculation, and only five showed intermediate-level symptoms and less viral load. The high susceptibility, observed across this collection representative of the cucumber Spanish diversity, reveals that ToLCNDV represents a major threat to cucumber cultivation. 

The cucumber accessions of other origins showed variable results. All accessions from China, and the single accessions from Japan, Sri Lanka, Iran, the United States, and D.R. Congo used in this study were susceptible to ToLCNDV. Interestingly, we have identified resistance in Indian accessions. CGN22297 and CGN22986 showed variable responses in symptom development and in viral load, suggesting that the resistance was not fixed in these accessions. The accessions CGN23089, CGN23423, and CGN23633 were uniformly resistant, symptomless, and had very low ToLCNDV accumulation compared to susceptible controls.

Finding virus resistance in *C. sativus* is not unexpected as this species has often been used as a source of resistance for different cucurbit viruses. For example, resistance genes to different potyviruses have been identified mainly in three cucumber accessions: ‘Suriman’, ‘Taichung Mou Gua’ (TMG-1), and ‘Dina-1′ [40]. In the inbred cucumber line ‘02245′, one *locus* controlling resistance to papaya ring spot virus (PRSV) and another controlling resistance to *Watermelon mosaic virus* (WMV), both recessives, were found by Tian et al. [41,42]. In the same line, resistance to the cucumovirus *Cucumber mosaic virus* (CMV) is quantitatively inherited [43] and in *C. sativus* var. *hardwickii*, Munshi et al. [44] identified CMV resistance controlled by a single recessive gene. Additionally, resistance to *Cucumber vein yellowing virus* (CVYV) has been reported in the Spanish landrace C.sat-10 [45], to *Cucurbit yellow stunting disorder virus* (CYSDV) controlled by more than one recessive gene [46], and in two Indian accessions of *C. sativus* to *Cucumber green mottle mosaic virus* (CGMMV) [47].

To date, most of the sources of resistance identified in cucurbits against ToLCNDV come from India. For instance, resistance to sponge gourd was identified in germplasm collected from different regions in India [17]. A dominant allele was found controlling the resistance [23]. In *Cucumis melo*, resistance to ToLCNDV was found in three accessions of the *momordica* horticultural group and two accessions of the wild *agrestis* group, all from India [19]. Finally, in *Cucurbita moschata*, genetic resistance to ToLCNDV has been identified in five accessions from different origins, one of them from India [25,48]. The fact that most of the ToLCNDV-resistant cucurbit accessions come from India could be related to the co-evolution of host and pathogen in this part of the world where ToLCNDV was detected infecting cucurbits many years ago [49]. 

The analysis of the F_1_ generation derived from the resistant accession CGN23089 suggests that the resistance to ToLCNDV found in cucumber is recessive. It is interesting to note that recessive control of resistance is frequent in several virus resistance systems. Recessive resistance genes interfere the viral life cycle at different levels: single cells, cell-to-cell movement, long-distance transport through the plant, and/or preventing high levels of virus accumulation [50]. In cucumber, the mechanism of resistance to ToLCNDV is characterized by a drastic and significant reduction of virus titer and infected plants are asymptomatic or exhibit mild disease symptoms. This type of resistance is similar to that observed in the rest of the resistances identified in the pathosystem of ToLCNDV–host. In cucurbits, the high level of ToLCNDV DNA accumulation in plant tissue results in the development of severe symptoms and leads to a major reduction in yield in the case of susceptible cultivars, but this does not happen for the cultivars showing resistance. The virus DNA level remains low and approximately constant and has minimal effects on the yield and health of plants [17,19,23,24,25,26]. In tomato, ToLCNDV viral DNA also determined the level of resistance and yield loss in test varieties of tomato under the same environmental conditions. Resistant cultivars showing a low level of viral DNA in their tissue when compared to other susceptible cultivars have been reported previously [51]. This also happens in the case of CGMMV in cucumber [47], CYSDV and WMV [52,53], and *Papaya ringspot virus* (PRSV) in squash and watermelon [54]. Further studies will be needed to establish the mechanism that limits ToLCNDV accumulation in resistant plants.

The accessions identified in this study are good candidates for breeding programs to avoid damage caused by ToLCNDV in *C. sativus*. Given the importance of ToLCNDV and the scarcity of sources of resistance to ToLCNDV in cucumber, the virus resistance found in accessions CGN23089, CGN23423, and CGN23633 should be introgressed into commercial cultivars. Our inheritance analyses indicate that the resistance to ToLCNDV in the CGN23089 accession is mainly controlled by one recessive gene, and this was supported by the detection of one QTL in chromosome 2 of the *C. sativus* genome. Despite the fact that this region was significantly linked to symptom development and viral load of ToLCNDV in cucumber, the percentage of phenotypic variance explained by the QTL (*R^2^*) is moderate. A higher density of the SNP panel covering the whole genome, along with a finer mapping of the candidate region, might likely increase this percentage. Nevertheless, the results obtained here, even with a small number of markers, contribute significantly to obtaining preliminary information about the *locus* implicated in ToLCNDV resistance in cucumber, and are in accordance with previous studies of genetic control of resistance to ToLCNDV in cucurbits. In melon, a major *locus* in chromosome 11 and two additional regions in chromosomes 2 and 12 controlling the resistance of the wild *agrestis* accession WM-7 were found [24]. In a recent publication, Romay et al. [55] identified in the same Indian accession WM-7 one recessive (*bgm-1*) and two dominant (*Bgm-2* and *Tolcndv*) genes controlling the resistance to ToLCNDV. In *Cucurbita moschata*, a major recessive gene located in chromosome 8 was found controlling the resistance in an Indian accession. This candidate region of *C. moschata* is syntenic to the region responsible for ToLCNDV resistance in chromosome 11 of melon [26]. Since both *loci* for resistance to ToLCNDV are syntenic and share a common cluster of genes, we looked for this cluster in synteny with the cucumber genome, which was located in chromosome 6 (from 6,527,862 pb to 6,756,572 pb genomic positions) (Table S2). Two SNPs used in this work are close to this region (SNPCs6_8 and SNPCs6_7, at 6,705,461pb and 7,276,564 pb, respectively), but none of them was significantly associated with the resistance to ToLCNDV. Thus, the candidate region identified here may be in a different region, associated with different resistance genes. Among the list of annotated genes in the candidate region of cucumber chromosome 2 (Table S3), there are three LRR receptor-like serine/threonine-protein kinases (CsGy2G012160, CsGy2G015920, and CsGy2G016150) implicated in resistance to ToLCNDV and other geminiviruses [56,57,58,59], four NAC domain transcription factors (CsGy2G015830, CsGy2G016100, CsGy2G016110, and CsGy2G016220), gene family associated with an increase in tomato plant susceptibility during ToLCNDV infection and resistance to a begomovirus in common bean (*Phaseolus vulgaris* L.) [60,61], and an RNA-directed DNA methylation protein (CsGy2G016290.1), one of the components of the RNA silencing pathway used against plant viruses in the defense response [62]. More interestingly, a 26S proteasome non-ATPase regulatory subunit 12 (CsGy2G015260) is included in this region. In tomato, a 26S proteasomal subunit RPT4a (SlRPT4) interferes with the genome transcription of ToLCNDV and induces the hypersensitive response [63]. Although SlRPT4 protein has an active ATPase activity, a possible effect of CsGy2G015260 against ToLCNDV infection must be further explored.

Our first approximation of candidate genes for resistance to ToLCNDV is being broadened with new sequencing assays, which will provide new molecular markers to finely map the identified QTL and facilitate marker-assisted breeding for ToLCNDV resistance in cucumber.

## 5. Conclusions

In this paper, germplasm accessions of cucumber (*Cucumis sativus*) from different geographical origins were screened for resistance to ToLCNDV. Three Indian accessions (CGN23089, CGN23423, and CGN23633), as well as all plants of their progenies obtained by selfing, were highly resistant to the mechanical inoculation, and remained symptomless and showed a reduced viral accumulation. Plants of the CGN23089 accession were crossed with plants of the susceptible accession BGV011742, and F_1_ hybrids were used to construct segregating populations (F_2_ and backcrosses), which were genotyped with SNPs distributed along the *C. sativus* genome. The results suggest a monogenic recessive genetic control, and a QTL in chromosome 2 of cucumber was identified controlling the resistance. The described SNPs linked to the resistance can be used in breeding programs to obtain cucumber cultivars with tolerance to ToLCNDV.

## Figures and Tables

**Table 1:** Response of Spanish landraces of *C. sativus* to the mechanical inoculation with ToLCNDV. Mean and range of symptoms scored in plants per genotype (at 15 and 30 dpi) according to the scale: 0, absence of symptoms; 1, mild symptoms; 2, moderate symptoms; 3, severe symptoms; 4, very severe symptoms or plant death. Mean score of viral load detected in each plant of the assayed accessions by tissue printing at 15 dpi according to the scale of high (+++), intermediate (++), low (+), or absent (-) viral accumulation. Data not available are shown as n/a.

			Symptoms at 15 Dpi		Symptoms at 30 Dpi		Viral Load
Type	Genebank Code	Spanish Province	Mean	Range	Mean	Range	Tissue Printing
Short	BGV000047	Zaragoza	1.2	(1–2)	1.8	(1–2)	+++
	BGV000408	Cádiz	1.6	(1–2)	1.6	(1–3)	+++
	BGV000437	Jaén	1.2	(0–3)	1.8	(1–2)	++
	BGV000467	Jaén	1.0	(0–2)	1.4	(0–3)	+++
	BGV000479	Córdoba	1.0	(1)	1.0	(0–2)	+++
	BGV000512	Huelva	1.0	(0–2)	1.4	(1–2)	+++
	BGV002495	Tenerife	n/a		0.6	(0–2)	++
	BGV003714	Cuenca	2.0	(1–3)	1.7	(1–2)	+++
	BGV004026	Cáceres	1.4	(1–2)	2.5	(1–3)	+++
	BGV004304	Murcia	n/a		2.0	(1–3)	+++
	BGV008299	Valencia	2.0	(1–3)	2.4	(2–3)	++
	BGV010301	Guadalajara	1.4	(0–3)	1.6	(0–3)	+++
	BGV010314	Guadalajara	1.7	(1–2)	1.2	(0–2)	+
	BGV010636	Soria	2.4	(1–4)	3.4	(2–4)	++
	BGV011582	Teruel	0.8	(0–2)	1.8	(1–2)	++
	BGV011734	Valladolid	2.0	(0–4)	3.2	(3–4)	++
	BGV011736	Ávila	2.6	(0–4)	2.8	(2–4)	++
	BGV011742	Albacete	3.4	(3–4)	3.8	(3–4)	++
	BGV014959	Huesca	3.8	(3–4)	3.6	(3–4)	++
	BGV015469	Cáceres	1.6	(0–2)	2.8	(2–4)	+
Long	BGV000372	Granada	0.6	(0–1)	1.2	(0–2)	++
	BGV000381	Málaga	0.6	(0–1)	2.4	(2–3)	+++
	BGV000416	Cádiz	1.4	(0–2)	2.0	(1–3)	++
	BGV001310	Asturias	1.0	(0–2)	1.6	(1–2)	+++
	BGV002494	Tenerife	2.0	(2)	1.0	(0–2)	+++
	BGV004305	Murcia	1.6	(1–2)	1.2	(1–2)	+++
	BGV004309	Murcia	1.4	(1–2)	3.0	(3)	+++
	BGV004851	Castellón	0.0	(0)	1.0	(0–2)	++
	BGV004926	Valencia	2.0	(2)	2.0	(2)	+++
	BGV004936	Valencia	1.4	(1–2)	1.3	(1–2)	++
	BGV011586	Orense	0.6	(0–3)	0.6	(0–3)	+
	BGV011724	Teruel	1.8	(0–4)	1.8	(0–4)	+
	BGV014967	Guadalajara	2.2	(0–4)	2.4	(1–4)	++
	BGV015229	Vizcaya	2.5	(1–3)	2.5	(2–3)	++
	BGV015696	Alicante	2.4	(0–4)	2.6	(0–4)	++
	BGV015700	Girona	2.8	(1–4)	3.4	(2–4)	++
French	BGV010290	Granada	2.8	(0–4)	3.8	(3–4)	+++
	BGV011735	Zaragoza	2.3	(0–3)	2.3	(0–4)	++
	BGV014961	Castellón	3.0	(0–4)	2.6	(0–4)	+++
	BGV014969	Cantabria	1.6	(0–3)	2.4	(0–4)	+++

**Table 2:** Response of *C. sativus* accessions from different origins to the mechanical inoculation with ToLCNDV. Mean and range of symptoms scored in plants per genotype (at 15 and 30 dpi) according to the scale: 0, absence of symptoms; 1, mild symptoms; 2, moderate symptoms; 3, severe symptoms; 4, very severe symptoms or plant death. Mean score of viral load detected in each plant of the assayed accessions by tissue printing at 15 dpi according to the scale of high (+++), intermediate (++), low (+), or absent (-) viral accumulation. Data not available are shown as n/a.

				Symptoms at 15 Dpi		Symptoms at 30 Dpi		Viral Load
Type	Genebank Code	Country	Local Name	Mean	Range	Mean	Range	Tissue Printing
Short	CGN19748	India	Khira	3.5	(3–4)	3.0	(3)	++
	CGN19817	India	Cucumber Medium	1.4	(0–3)	3.0	(2–4)	+++
	CGN20512	Netherlands	752	2.5	(2–3)	2.8	(2–3)	++
	CGN20517	Sri Lanka	Yellow 1	1.0	(0–2)	1.3	(0–2)	+
	CGN21585	India	Saharanpur	0.3	(0–1)	2.3	(1–4)	n/a
	CGN21691	D.R. Congo	N2/81	2.8	(0–4)	3.2	(1–4)	+++
	CGN22280	India	Shuei Huang Kua	1.0	(0–3)	1.0	(1)	+++
	CGN22986	India	Smallgreen	0.4	(0–1)	0.4	(0–1)	++
	CGN23089	India	Anthracnose 197087	0.2	(0–1)	0.0	(0)	-
	CGN23411	India	Khira Cheshuicchatyi	0.6	(0–2)	1.0	(0–3)	++
	CGN23423	India	JL-2 Dhillon	0.0	(0)	0.3	(0–1)	-
	CGN23633	India	Jaipur Balam	0.0	(0)	0.7	(0-1)	-
Medium	CGN19819	India	Puneri Klura	0.8	(0–1)	1.3	(1–2)	+++
	CGN20853	Japan	Sagami Hanpaku Fushinari Kyuri	1.5	(0–2)	1.5	(1–2)	+++
	CGN21616	Iran	Rasht	3.3	(2–4)	3.7	(3–4)	+++
	CGN22281	India	Long Green	0.8	(0–2)	1.5	(0–2)	++
	CGN22297	India	K-75	0.4	(0–1)	0.8	(0–2)	+
Long	BGV015107	China	Hei Wu She	1.2	(0–2)	1.6	(1–3)	++
	BGV015113	China	Shou Guang Qiu Gua	2.0	(1–3)	1.6	(1–2)	++
	BGV015115	China	Long Quan Qing Huang Gua	1.6	(0–4)	1.6	(1–3)	++
	BGV015116	China	De Hui Huang Gua	2.0	(0–3)	2.6	(1–4)	+++
	BGV015118	China	San Ye Zao	2.5	(0–4)	3.3	(1–4)	+++
-	CGN19655	U.S.A.	SC 53-B (6)	1.4	(0–4)	2.4	(0–4)	+++

**Table 3:** Number of resistant and susceptible plants in each segregating population according to symptom development and ToLCNDV titers. The probability of *X*^2^ value was calculated for the expected ratio of one recessive gene controlling the resistance.

Populations	Resistant	Susceptible	Expected Frequencies	*X* ^2^
**F_2_**	31	65	1:3	2.722 (*p* = 0.0990)
**BC_CGN23089_**	21	33	1:1	2.667 (*p* = 0.1025)
**BC_BGV011742_**	0	11	-	-

**Table 4:** Quantitative trait loci (QTLs) identified in the F_2_ segregating population using composite interval mapping (CIM) and Kruskal–Wallis tests.

Trait	Chr ^a^	Nearest Marker ^b^	CIM					Kruskal–Wallis	
			Interval ^c^ (cM)	Add Effect ^d^	Dom Effect ^e^	LOD ^f^	*R^2^* ^g^	K* ^h^	Significance ^i^
Symptoms 30 dpi	2	SNPCs2_3	28–40	0.46	0.73	3.38	0.15	16.94	******
Viral load (Semiquantitative)	2	SNPCs2_3	24–32	0.18	0.79	3.07	0.14	13.01	****
Viral load (Quantitative, ΔΔCt)	2	SNPCs2_3	-	−1.45	−4.61	2.54	0.12	14.05	*****
Resistance (Qualitative trait)	2	SNPCs2_3	34–54	−0.23	−0.57	3.93	0.17	13.02	****

^a^ Chromosome; ^b^ the closest marker to the LOD peak, ^c^ interval position of the putative QTL, identified in the F_2_ (CGN23089-2 x BGV011742) by CIM, in cM on the genetic map; ^d^ *Add effect*: additive effect of the BGV011742 allele; ^e^ *Dom effect*: dominant effect of the BGV011742 allele; ^f^ *LOD*: higher logarithm of the odds score; ^g^ *R^2^*: percentage of phenotypic variance explained by the QTL; ^h^ K*: the Kruskal–Wallis test statistic; ^i^ Significance level in the Kruskal–Wallis test ****: 0.005, *****: 0.001, ******: 0.0005.

## Data Availability

Data in this study are available from the authors upon request.

## Supplementary Materials

The following are available online at https://www.mdpi.com/article/10.3390/microorganisms9050913/s1, Table S1: List of polymorphic SNPs in the F_2_ (CGN23089-2 x BGV011742) population. Their position in the genome is according to Version 2 of the cucumber genome Gy14 (http://cucurbitgenomics.org). The positions in the genetic map were calculated using Kosambi’s function and used for QTL analysis. Table S2: Syntenic genes between candidate region for ToLCNDV resistance in chromosome 8 of *C. moschata*, chromosome 11 of *C. melo*, and chromosome 6 of *C. sativus* genomes (v1.0, v3.6.1, and Gy14-v2, respectively), determined with Tripal “SyntenyViewer”, available at cucurbitgenomics.com. Table S3: Annotated genes in the candidate region of chromosome 6 of *C. sativus* genome (Gy14-v2) available at cucurbitgenomics.com.
